# Ultrahigh Dimensional Variable Selection for Interpolation of Point Referenced Spatial Data: A Digital Soil Mapping Case Study

**DOI:** 10.1371/journal.pone.0162489

**Published:** 2016-09-07

**Authors:** Benjamin R. Fitzpatrick, David W. Lamb, Kerrie Mengersen

**Affiliations:** 1 Mathematical Sciences School, Queensland University of Technology (QUT), Brisbane, QLD 4001, Australia; 2 Cooperative Research Centre for Spatial Information (CRCSI), Carlton, VIC 3053, Australia; 3 Institute for Future Environments, Queensland University of Technology (QUT), Brisbane, QLD 4001, Australia; 4 Precision Agriculture Research Group, University of New England, Armidale, NSW 2351, Australia; 5 ARC Centre of Excellence for Mathematical and Statistical Frontiers, Queensland University of Technology (QUT), Brisbane, QLD 4001, Australia; University of Missouri Columbia, UNITED STATES

## Abstract

Modern soil mapping is characterised by the need to interpolate point referenced (geostatistical) observations and the availability of large numbers of environmental characteristics for consideration as covariates to aid this interpolation. Modelling tasks of this nature also occur in other fields such as biogeography and environmental science. This analysis employs the Least Angle Regression (LAR) algorithm for fitting Least Absolute Shrinkage and Selection Operator (LASSO) penalized Multiple Linear Regressions models. This analysis demonstrates the efficiency of the LAR algorithm at selecting covariates to aid the interpolation of geostatistical soil carbon observations. Where an exhaustive search of the models that could be constructed from 800 potential covariate terms and 60 observations would be prohibitively demanding, LASSO variable selection is accomplished with trivial computational investment.

## 1 Introduction

Global soils have been estimated to contain the largest pool of terrestrial organic carbon in the biosphere, storing more carbon than all land plants and the atmosphere combined [1]. The importance of the dynamic equilibrium between carbon in soils and carbon in the atmosphere has been illustrated by such estimates as there having been 3.3 times the amount of carbon in the atmosphere as *CO*_2_(g) present in global soils [2]. More than half of the global soil carbon pool has been estimated to be comprised of organic compounds collectively referred to as soil organic carbon (hereafter SOC) [2]. SOC may be depleted to as little as 25% of capacity when natural ecosystems are converted into agricultural systems with the majority of this carbon lost to the atmosphere as *CO*_2_(g) [2]. The contribution such SOC losses would have made to terrestrial carbon dynamics may be appreciated in the context of the estimate that 34% of the global land surface had been devoted to agriculture by 2007 [3]. Recharging SOC levels by sequestering *CO*_2_(g) in agricultural soils has been demonstrated to provide direct benefits to agriculture, in addition to providing an opportunity to partially offset anthropogenic green house gas emissions [4]. Consequently, it is a key feature of national and international carbon accounting endeavours.

The effort and cost associated with sampling SOC via laboratory analysis of soil core samples has led to a need to improve soil core sample based maps of SOC through statistical modelling using more readily attainable environmental variables as covariates. Covariates are also referred to as explanatory or independent variables. Improving predicted maps by using environmental variables as covariates in the models that produce these maps is common in modern soil carbon modelling [5–10]. S1 Table also summarizes some of the diversity of soil carbon modelling studies that have been completed to date globally. Predicting quantitative maps of soil characteristics from empirical data has been referred to as digital soil mapping [11, 12]. This task has been characterized by limited numbers of geostatistical (spatial point referenced) observations of the response variable [12] (the variable a model predicts, also referred to as the dependent variable) and much finer resolution geostatistical data and or full cover areal data on diverse collections of environmental characteristics of potential relevance as covariates for modelling the response, again see S1 Table for examples. As such, the methodological challenges of digital soil mapping bear marked similarities to those encountered in other fields where a set of ‘ground truthed’ geostatistical or non-contiguous areal observations (plots or quadrats) are sought to be interpolated with the aid of other environmental data available across the area on interest. Examples of this analysis task outside soil science include modelling above ground biomass in forests [13] and semi-arid regions [14] along with species distribution modelling and biogeography [15]. In each case a model is built from some collection of environmental characteristics to interpolate and or extrapolate from a set of response observations. Such modelling is often accompanied by two challenges. The first is spatial misalignment of observations of different variables and or observations and the locations (or coverage extents and resolutions) to which the response variable is to be interpolated. This challenge is recognised in statistics and methods exist to address it [16, 17]. The second is the availability of large numbers of potentially relevant covariates coupled with the belief that some of these covariates will be more useful for predicting the response variable (soil carbon in this study) than others. Selection of a subset of available covariates for use in a model is variously referred to as variable selection and subset selection. This is a broad area of statistics that overlaps with the area of machine learning concerned with finding sparse solutions to supervised learning problems [18]. In this paper, spatial misalignment between different variables in the data is addressed and the main focus is selection of a parsimonious subset of covariates to aid interpolation of the response variable under an ultrahigh dimensional scenario (the scenario where the number of covariates exceeds the number of observations [19]). This is achieved by showcasing the performance of Least Absolute Shrinkage Selection Operator (LASSO) penalized Multiple Linear Regression (MLR) models on data from a real world case study of soil core derived observations of %SOC across 137ha of agricultural land in New South Wales, Australia. The remainder of the article is structured as follows. Section 2 describes the field site along with the data collection, collation and spatial realignment for the case study. In Section 3 the motivation for the selection of LASSO variable selection is outlined and the key characteristics of this method are summarized. Section 4 contains the results and discussion of the analysis of the case study data. In Section 4, LASSO variable selection is compared to four popular variable selection methods in terms of the set of covariates selected and the predictive performance of the models selected. Section 4 also contains a description of fitting regression models using covariates calculated from the spatial coordinates of the observations of the response variable (spatial polynomial regressions) to the residuals from the environmental covariate based modelling for more precise interpolation of %SOC. The correction of predictions from the covariate based modelling of the response variable with predictions from regression models fitted to the residuals of this first round of modelling and production of a full cover predicted raster for %SOC is also described. Section 5 contains discussion of this work and promising avenues for future research.

## 2 Data Collection & Preparation

### 2.1 Data Collection & Collation

The case study data were collected from a 137ha area of native pasture with remnant woody vegetation on the Sustainable, Manageable, Accessible, Rural Technology (SMART) Farm of the University of New England near Armidale, New South Wales, Australia. The 60 observations of the response variable, percentage soil organic carbon (%SOC), include 57 values less than 2.55% while the remaining three values are 3.08%, 5.01% and 5.13%. The 63 environmental characteristics considered here as potential covariates are summarized in Table 1. The Digital Elevation Model (DEM) derived covariates (see Terrain and Hydrology metrics in Table 1) were calculated with the System for Automated Geoscientific Analyses (SAGA software v2.1.0) [20] and the rasters produced for each of these covariates were read into R [21] with the ‘RSAGA’ [22] package. The remaining raster covariates were read into R with the R package ‘raster’ [23]. Further details regarding the study site, field methodology and covariates are provided in Appendices A and B in S1 Appendices.

**Table 1:** The 63 potential covariates.

Source	Covariate Name	Acronym
ATV Top of Pasture Surveys 12 covariates from each of February, May & November = 36 covariates	Soil Apparent Electrical Conductivity	ECA
	Near InfraRed Reflectance	NIR
	Red Reflectance	RED
	Simple Ratio	SR
	Difference Vegetation Index	DVI
	Normalized Difference Vegetation Index	NDVI
	Soil Adjusted Vegetation Index	SAVI
	Non-Linear Vegetation Index	NLVI
	Modified Non-Linear Vegetation Index	MNLVI
	Modified Simple Ratio	MSR
	Transformed Vegetation Index	TVI
	Re-normalised Difference Vegetation Index	RDVI
Terrain & Hydrology Metrics Calculated from 25*m*^2^ resolution DEM = 16 Covariates	Catchment Area	CatAr
	Catchment Height	CatHe
	Catchment Slope	CatSl
	Cosine(Aspect)	CosAsp
	Elevation	Elev
	Slope Length Factor	LSF
	Plan Curvature	PlanC
	Profile Curvature	ProfC
	Sky View Factor	SVF
	Slope	Slp
	Stream Power Index	SPI
	Terrain Ruggedness Index	TRI
	Topographic Position Index	TPI
	Vector Terrain Ruggedness	VTR
	Visible Sky	VS
	Wetness Index	WI
Foliar Projective Cover Layers = 2 Covariates	2011	FPCI
	2012	FPCII
Electromagnetic Channels = 6 Covariates	1 to 6	MagI—MagVI
*γ* Radiometric Layers = 3 Covariates	Potassium	K
	Thorium	Th
	Uranium	U

### 2.2 Spatial Realignment of Covariate and Response Observations

The data used in this analysis consists of two types of spatial data: point referenced data and areal data [24]. Point referenced data are also referred to as geostatistical data [24]. The soil core derived %SOC observations and the covariates observed via the All Terrain Vehicle (ATV) survey (see Appendices A and B in S1 Appendices for more details on the ATV survey) are examples of geostatistical data from the case study. The areal data utilized in this work consists of observations of regular grids of rectangular pixels; such data are often referred to as raster data. The DEM derived covariates, the foliar projective cover layers and the *γ* ray radiometric survey data are all examples of raster data. The geostatistical observations of the response variable are available at one set of spatial point locations over the study area while the observations of the covariates available as geostatistical data are available at a separate set of spatial point locations over the same area. Thus the geostatistical covariate observations are spatially misaligned [17] from the geostatistical observations of the response variable. To model the observations of the response variable with these spatially misaligned covariate observations the covariates must first be interpolated to the locations at which the response variable was observed, thereby addressing a point to misaligned point class of change of support problem [16, 17]. There is also a change of support problem inherent in the use of the pixels of covariate rasters to predict the geostatistical observations of the response variable. In the terminology of Banerjee et al. [17] this involves a block to point class of change of support. Elegant methods exist to address these change of support problems via hierarchical approaches to regression (also referred to as multi-level modelling) [25]. As the primary objective in this work is exploration of variable selection methods to aid interpolation, the simpler approach of realigning the data to address the change of support problems encountered prior to conducting variable selection has been adopted. As the majority of the covariate rasters for the case study are derived from the 25m^2^ resolution DEM, all covariates are realigned to square 25m by 25m pixels centered on each observation of the response variable. Geostatistical covariates are interpolated to regularly spaced rectangular arrays of 100 by 100 points spanning these 25m by 25m square pixels via thin plate splines with the R package ‘fields’ [26]. The covariate value accompanying each observation of the response variable is calculated as the mean of the covariates values interpolated to the array centered on that observation of the response variable. The raster covariates are realigned to these 25m by 25m square pixels centered on each observation of the response variable by a similar process. In this process, the values of raster covariates are queried at these same rectangular arrays of 100 by 100 points that spanned the 25m by 25m square pixels centred on each observation of the response variable. The realigned value of each of these covariates to accompany each of the response observations is taken as the mean of the values of the covariate across the array of points centered on that observation of the response variable.

## 3 Statistical Background

### 3.1 Choice of Modelling Method

A variety of statistical and machine learning techniques have been applied to soil carbon modelling. Such techniques include ANOVA [27], multiple linear regression (MLR) [28], MLR with stepwise variable selection [5, 29–32], MLR on the principal components of the covariate observations [33], regression fitted by partial least squares [34], MLR with stepwise variable selection within groups of the data identified via neural networks [35] and regression kriging [7, 36]. Binary tree based methods applied to soil carbon modelling include Classification And Regression Trees (CART) [37, 38], Random Forests [38] and CUBIST [8–10, 39, 40]. The advantages and disadvantages of a range of statistical and machine learning techniques are evaluated in terms of the objective of covariate assisted interpolation, associated computational demands and appropriateness for application to data with the three defining characteristics of the case study data: (1) more potential covariate terms than observations (ultrahigh dimensionality) (2) a high degree of collinearity among the potential covariate terms and (3) suspected importance of non-linear effects of covariates and interactions of covariate effects. The MLR based approaches considered include: ridge regression [41], LASSO modified MLR fitted via quadratic programming [42], LASSO modified MLR fitted by the Least Angle Regression (hereafter LAR) algorithm [43] and the Bayesian LASSO [44]. The CART based techniques considered include: Bayesian CART [45], bagged regression trees [46], random forests [47], boruta all relevant variable selection [48], boosted regression trees [49], cubist [50] (https://www.rulequest.com/cubist-info.html) and Bayesian treed regression [51]. This evaluation is summarised in Appendix C in S1 Appendices.

The case study analysis is conducted with LASSO modified MLR fitted via the LAR algorithm. Model-averaging the predictions from the LASSO solutions obtained from LAR executions within a cross validation scheme yields an aggregate estimate in a manner similar to random forests, bagged trees and boosted trees. A cross validation based approach also facilitates estimation of the shrinkage parameter for the LASSO fits (*λ* in Eq 1). The choice of LASSO modified MLR allows the importance of covariate terms (linear, non-linear and interaction) to be compared in terms of which have coefficients that are shrunk to zero and which are assigned non-zero values. In contrast, whether the overall role of a covariate within the aggregated estimate from random forests, bagged or boosted trees is closer to linear or non-linear (and if non-linear what manner of non-linear) would be harder to judge from the results of such a fit. This ease of interpretability of the LASSO modified MLR comes with the cost of having to recenter and rescale (to mean zero and magnitude one) all covariates in each training set (a requirement of the LAR algorithm [43]) and mirror those transformations on each associated validation set. Whereas, such transformations are unnecessary for binary tree based techniques.

### 3.2 LASSO Variable Selection as a Special Case of PLS

Penalized Least Squares (PLS) coefficient estimates ($\hat{\beta }$ in Eq 1) are calculated by identifying the coefficient estimate vector that minimizes the sum of the residual sum of squares and the result of applying some penalty function to the coefficients. Simple PLS estimates use the *L*_*γ*_ norm $\sum_{j=1}^{p}{\left|\beta_{j}\right|}^{\gamma }$ of the coefficient vector ***β*** for some *γ* > 0 as the penalty function so that

$$\hat{\beta }=\underset{\beta }{arg min}\{\sum_{i=1}^{n}{\left(y_{i}-\beta_{0}-\sum_{j=1}^{p}x_{ij}\beta_{j}\right)}^{2}+\lambda \sum_{j=1}^{p}|\beta_{j}{|}^{\gamma }\},\;\;\gamma >0$$ (1)

where the tuning parameter *λ* controls the degree to which $\hat{\beta }$ is shrunk towards the zero vector [52]. When *γ* is set to 1, the solution to Eq 1 is the *L*_1_ PLS estimate of ***β***, also known as the Least Absolute Shrinkage and Selection Operator (LASSO) [42]. When *γ* is set to 2, the solution to Eq 1 is the *L*_2_ PLS estimate of ***β*** which is referred to as a ridge regression estimate [41]. Other penalized least squares techniques including adaptive LASSO [53], Smoothly Clipped Absolute Deviation (SCAD) [54] and Minimax Concave Penalty (MCP) [55] are derived through use of more complex penalty functions in place of the *L*_*γ*_ norm in Eq 1. Solving Eq 1 with *γ* set to a value of 2 or less results in the values of some coefficients being estimated as zero exactly (how many depends on the value of the tuning parameter *λ*) [52]. Since a coefficient estimate of zero is equivalent to exclusion from the selected model such a solution effectively performs both variable selection and shrinkage. As such, *L*_*γ*_ penalized estimation with *γ* < 2 is applicable to the case study where the number of potential covariates exceeds the number of observations (*p* > *n*).

The requirement for a computational solution to *L*_1_ penalized estimation (stemming from the presence of the absolute value in Eq 1) was originally addressed via relatively computationally expensive quadratic programming [42] and has been addressed more recently by the computationally efficient Least Angle Regression (LAR) algorithm [43]. From the PLS family of techniques, *L*_1_ penalized estimation has been selected for use in the case study analysis for three reasons: 1)] suitability for variable selection and modelling with correlated covariates, 2) suitability for variable selection in scenarios with *p* > *n*, and 3) the computational efficiency of the LAR algorithm [43].

The LAR algorithm has been designed such that covariates continue to be added to the model until either the available degrees of freedom are exhausted or there are no covariates outside the current model that have a correlation with the current residual vector greater in magnitude than some user-specified threshold value. In the case of the LASSO modification of the LAR algorithm, while steps of the algorithm may result in a covariate being removed from the current model, the algorithm still proceeds to add and remove covariates from the current model until either of the above criteria are met. Subsequently, the LAR algorithm (and the LASSO variant thereof) returns a sequence of selected models from which it is necessary to choose a parsimonious final model. Efron et al. [43] derive a *C*_*p*_ style stopping criterion for the LAR algorithm but note that this is most appropriate in scenarios with less potential covariates than observations. Alternative stopping criteria, applicable to more general scenarios, also exist [56] though cross validation is a popular approach for ultrahigh dimensional problems [57–59]. Hence, a cross validation based approach to making the final selection from the sequence of selected models produced by the LAR algorithm is adopted here. All analysis is conducted in the R language and environment for statistical computing [21] and all graphics are produced with the R package ‘ggplot2’ [60]. The data and R code associated with this work are provided via a repository located at https://github.com/brfitzpatrick/larc.

## 4 Methods and Results

### 4.1 Comparison of Variable Selection Methods for MLR

LASSO variable selection is compared to the more generic variable selection methods: exhaustive search, forward stepwise selection, backwards stepwise selection and sequential replacement selection (also known as stepwise forwards-backwards variable selection) on the case study data. Due to the complexity of interacting processes that may influence the formation, distribution and loss of SOC across the study site, polynomial terms up to order four for each covariate and all possible pairwise interactions of the covariates are considered. The full set of potential covariates thus expands from 63 to 2205 potential covariate terms (63*4+(632)). With 60 observations of the response variable, if it were desired to explore all possible models from an intercept only model up to those that used the available degrees of freedom, some ∑i=160(2206i)≈2.27*10118 different models would need to be fitted and compared in an exhaustive search. To reduce the number of covariates considered and thus the required breadth of exhaustive search, the design matrix (the matrix of the covariate observations organised such that the covariate observations associated with particular response observations form the rows of the matrix and the observations of each covariate forms the columns of the matrix) is filtered to ensure that no remaining pairs of covariates have correlation coefficients greater in magnitude than some critical value. Since the correlation of a potential covariate with the response variable may be a poor indicator of the explanatory utility of this covariate in the presence of other covariates, the selection of covariates to retain from highly correlated pairs of covariates is based upon the spatial resolution at which each covariate is available. The motivation behind this decision being an effort to optimise the spatial accuracy of the interpolation of the response variable. For covariates with the same spatial resolution, the one derived from the simpler function of observed data is chosen, otherwise the choice is made at random. These criteria are discussed in more detail in Appendix D in S1 Appendices.

Filtering the design matrix to enforce a maximum correlation coefficient magnitude (hereafter MCCM) of 0.4 between remaining covariate pairs results in a design matrix with 27 covariate terms. The branch-and-bound algorithm implemented in the ‘leaps’ package [61] requires only a subset of the ∑i=128(28i)≈2.68*108 models it is possible to construct from this design matrix to be fitted in order to determine the optimal model that would be returned from a full exhaustive search [62]. The objective of building models for interpolation of the response variable motivated the decision to compare the results of the variable selection techniques trialed in terms of the ability of the models selected to predict data held out from the fitting process. These comparisons are conducted on 500 unique divisions of the data into training and validation sets in a cross validation scheme. This cross validation scheme uses training sets of 35 observations and validation sets of 25 observations. The selection of a training set size is discussed in Appendix E in S1 Appendices.

Training sets constructed from the design matrix composed of 27 covariate terms are supplied to each of the variable selection methods (LASSO variable selection, forward selection, backward selection, sequential replacement and exhaustive search variable selection). In each case the final selection from the sequence of models returned is made to minimise the validation set element prediction error (here after VSEPE) sum of squares. The distributions of VSEPE absolute values from each variable selection technique are summarized in Table 2. When applied to these austerely filtered design matrices, all five variable selection techniques yield very similar VSEPE distributions. The first three quarters of the ordered VSEPE absolute values obtained from LASSO variable selection are slightly more compressed towards zero than those from any other technique considered.

**Table 2:** Summary statistics for the absolute values of validation set element prediction error (VSEPE) distributions from each variable selection method conducted on design matrices filtered to enforce a maximum correlation coefficient magnitude between covariate pairs of 0.4 or 0.95 (|*r*| ≤ 0.4 or |*r*| ≤ 0.95). The final column contains the coefficient of determination (*R*^2^) values for the model-averaged predictions (MAP) from the models resulting from the combinations of variable selection technique and design matrix filtering austerity specified by that row. LAR = Least Angle Regression Variable Selection, Exh = Exhaustive Search Variable Selection, Seq = Sequential Replacement Variable Selection, Fwd = Forward Stepwise Variable Selection, Bwd = Backward Stepwise Variable Selection, Min. = Minimum, 1st Qu. = First Quartile, 3rd Qu. = Third Quartile and Max. = Maximum.

		VSEPE						MAP
Method	|*r*| ≤	Min.	1st Qu.	Median	Mean	3rd Qu.	Max.	*R*^2^
LAR	0.95	1.332e-05	0.1482	0.3184	0.4744	0.5446	4.437	0.5963
LAR	0.40	1.097e-05	0.1517	0.3324	0.4776	0.5695	4.063	0.3666
Exh	0.40	5.571e-05	0.1644	0.3419	0.4964	0.5997	4.290	0.2882
Seq	0.40	5.571e-05	0.1677	0.3448	0.4960	0.6044	3.961	0.3055
Fwd	0.40	5.571e-05	0.1604	0.3392	0.4955	0.5994	4.063	0.3046
Bwd	0.40	1.036e-05	0.1654	0.3593	0.5053	0.6037	4.422	0.2382

Given a collection of models, a model-averaged prediction of an observation is the average of the predictions from each of these models of that observation. In this case the collection of models is comprised of the models selected for the 500 training sets and the averages computed are weighted averages. Predictions from the 500 selected models (one per training set) are model-averaged with weights inversely proportional to the prediction error sum of squares on the associated validation sets. Taking *i* to index the 500 divisions of the data into training and validation sets, the weights for model-averaging, *W*_*i*_, are calculated following Eq 2. Here *e*_*i*, *j*_ is the prediction error of the *j*^th^ element of the *i*^th^ validation set where each validation set has *v* elements.

$$W_{i}=\frac{\frac{1}{\sum_{j=1}^{v}e_{i,\;j}^{2}}}{\sum_{i=1}^{500}\frac{1}{\sum_{j=1}^{v}e_{i,\;j}^{2}}}$$ (2)

The noticeable improvement in accuracy of the model-averaged predictions from the models selected by LAR is shown in the column of coefficient of determination values in Table 2. Corresponding summary statistics for the absolute values of the VSEPE obtained from model fitted to 800 term design matrices that result from using a much less stringent MCCM of 0.95 are also included in Table 2 along with the coefficient of determination for the associated model-averaged predictions. Similar improvements, with greater magnitude, are observed between the LAR selected models for the 27 covariate design matrices and the 800 covariate design matrices as were observed between models selected by other variable selection techniques and LAR selected models. These improvements come with an increased computational cost, but application of the LAR algorithm to these expanded design matrices is still feasible requiring 21 minutes on a mid range laptop computer run to completion on all 500 training sets (an average of 2.52 seconds per training set). In contrast, exhaustive search variable selection on these expanded design matrices would be infeasible. The positive outliers in all the VSEPE distributions are likely the result of the three positive outliers in the observations of the response variable. When these are drawn as members of a validation set, models built from the associated training set likely under-predict these values in the validation set.

**Fig 1:**
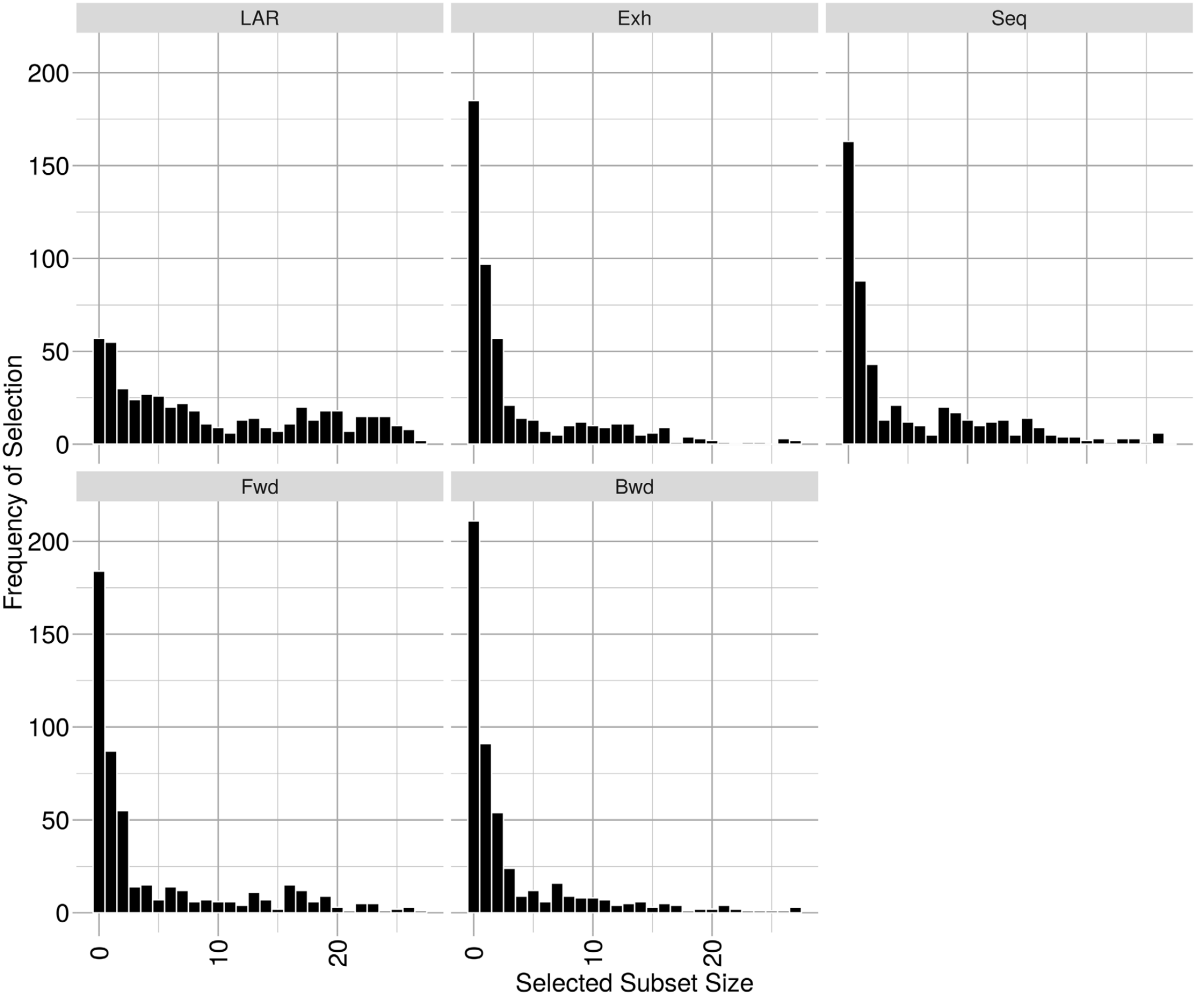
Histograms depicting the distribution of subset sizes selected by each variable selection technique applied to training sets constructed from the 27 covariate design matrix. LAR = Least Angle Regression Variable Selection, Exh = Exhaustive Search Variable Selection, Seq = Sequential Replacement Variable Selection, Fwd = Forward Stepwise Variable Selection, Bwd = Backward Stepwise Variable Selection, Min. = Minimum, 1st Qu. = First Quartile, 3rd Qu. = Third Quartile and Max. = Maximum.

The distributions of the numbers of covariates selected by each of the variable selection methods from the 27 covariate design matrices are depicted in Fig 1. The LASSO method results in intercept only models far less frequently and larger numbers of covariates per model more frequently than the other techniques. The differences in predictive accuracy and numbers of covariates selected per model, between the LASSO and the forwards stepwise OLS based method may be explained in terms of the comparative theoretical properties of these algorithms. At each step in the respective algorithms, both approaches choose the covariate most correlated with the current residual vector for inclusion in the current model. However, LAR adds this new covariate to the model in such a manner that the resulting prediction vector is equiangular between the previous prediction vector and this new covariate vector and only proceeds along this new prediction vector until some other covariate outside the current model is as correlated with the current residual vector as the most recently added covariate before repeating this procedure. Forwards selection, backwards stepwise variable selection and sequential replacement variable selection lack this facility to compromise between the correlated covariates. Furthermore, the differences between the results of LASSO variable selection and the exhaustive search variable selection may well stem from exhaustive search variable selection using OLS model fitting while the LASSO variable selection uses PLS based model fitting.

### 4.2 Frequently Selected Covariates

**Fig 2:**
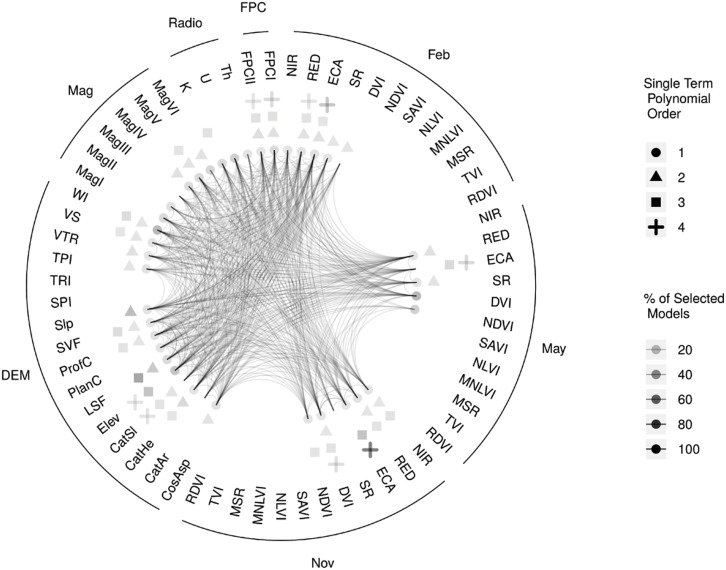
The frequencies with which covariate terms were selected across 500 selected models. These selected models were obtained by applying the Least Angle Regression variable selection algorithm to training sets constructed by taking 35 observation subsets of a design matrix. This design matrix was produced by filtering the full design matrix to enforce a maximum permitted correlation coefficient magnitude between covariate pairs of 0.95. The curved lines (Poincaré segments) represent interaction terms between the covariates they connect. Covariate acronyms are expanded in Table 1.

The numbers of the 500 selected models in which particular covariate terms occur can serve as an indicator of the relative importance of these terms for predicting the observations of the response variable. Table 3 lists the 15 most frequently selected terms from LAR variable selection on the 800 column design matrices. Table 3 also lists covariate terms from the 2205 column design matrix which were very highly correlated (|*r*| > 0.95) with these top 15 covariates and were thus excluded from the analysis in the design matrix filtering step. A chord diagram depicting the selection frequencies of all 800 covariate terms is presented in Fig 2. The complexity of interacting processes producing the spatial distributions of SOC in agricultural landscapes like that of the case study site is reflected in the diversity of the categories of covariates terms selected (soil *EC*_*a*_, vegetation indices, DEM derived metrics, magnetic imagery, radiometric imagery and foliar projective cover layers) and the mixture of linear terms, higher order polynomial terms and interactions of linear terms selected for these covariates.

**Table 3:** The 15 most frequently selected covariates from the LAR variable selection executions on the 500 unique, 35 observation training sets constructed from the design matrix created by filtering the full design matrix to enforce a maximum permitted correlation coefficient magnitude between remaining covariates pairs of 0.95. The second column contains the frequencies with which the selected covariates occurred in the 500 selected models. Accompanying each selected covariate in the final column are the covariates from the full design matrix that had correlation coefficient magnitudes with the covariate in question greater than 0.95 and thus were excluded from the design matrix supplied to the variable selection. Colons denote interaction terms for the two covariate terms which the colon separates. Numeric superscripts denote polynomial terms for the covariate indicated by the acronym. Acronyms are expanded in Table 1.

Covariate	Freq	Correlated Covariates
ECA.Nov^4^	219	-
LSF^3^	139	Slp^3^, TRI^3^, LSF^4^, Slp^4^, TRI^4^
DVI.May	102	SAVI.May, NLVI.May, MNLVI.May, RDVI.May
WI	100	-
ECA.Feb:Slp	95	ECA.Feb:TRI
Mag.II:FPCI	95	-
SVF:Mag.IV	94	-
Slp^2^	89	LSF:Slp, LSF:TRI, Slp:TRI, TRI:WI, TRI^2^
ECA.Feb:SR.May	88	ECA.Feb:NDVI.May, ECA.Feb:SAVI.May, ECA.Feb:MSR.May, ECA.Feb:TVI.May, ECA.Feb:RDVI.May
LSF:SVF	82	LSF:VTR, SVF:Slp, SVF:TRI
ECA.Nov:DVI.Nov	78	ECA.Nov:MNLVI.Nov
Elev:SVF	76	-
ECA.Feb:DVI.Nov	74	ECA.Feb:MNLVI.Nov, ECA.Feb:RDVI.Nov
ECA.Nov^3^	73	-
ECA.Feb:Elev	72	-

### 4.3 Modelling Spatial Component of Error

Following the model-averaging described above, regression models are fitted to the residual %SOC variation at each soil core location using a set of explanatory variables comprised of polynomial and interaction terms calculated from the spatial coordinates of each soil core observation. This allows spatial position to serve as a locally appropriate proxy for all the unobserved processes and interactions that may influence the spatial distribution of %SOC at the case study site. An alternative approach would be to use Kriging to spatially interpolate the residuals, but this requires the comparison of numerous pairs of orthogonal, directional, empirical semivariograms. A more attractive alternative is to calculate an empirical semivariogram raster, in which pairwise differences between geostatistical observations are assigned to two dimensional displacement bins and the empirical semivariance is calculated for each bin. The resulting raster may then either be smoothed [25] or simply examined directly and the spatial symmetry of the resulting values considered. However, the small sample size in the case study data would result in moderate numbers of pairs per bin only when a relatively large bin size is used. The resulting coarse spatial resolution would make characterisation of any detected anisotropy infeasible. Thus a simpler approach of fitting spatial polynomial regression models to the residuals and model-averaging the results via the same procedure used for the covariate based modelling is adopted.

The computational efficiency of the LAR algorithm enables us to explore design matrices that include single term polynomials for Easting and Northing values up to polynomial order 12 and interaction terms constructed from subsets of these single term polynomials such that all possible product terms which equate to an overall polynomial order of 6 or less are included in this exploration. Interaction terms considered range from pairwise interaction terms to interaction terms equivalent to a polynomial term of half the order of the maximum order of single polynomial terms considered. This limit is imposed to avoid confounding between interaction terms of order equivalent to the higher order single polynomial terms. The results of fitting spatial polynomial regression models to training sets of 35 observations constructed from the design matrix filtered to enforce a MCCM between covariate pairs of 0.95 are used for similar reasons involved in this decision for the covariate based variable selection. Again, 500 unique divisions of the data into training and validation sets are constructed and explored by LAR variable selection and final selections are made from each LAR model choice trajectory on the basis of which model minimizes the associated VSEPE sum of squares. Model-averaging is conducted with weights inversely proportional to the VSEPE sums of squares as per Eq 2.

### 4.4 Full Cover Inference

**Fig 3:**
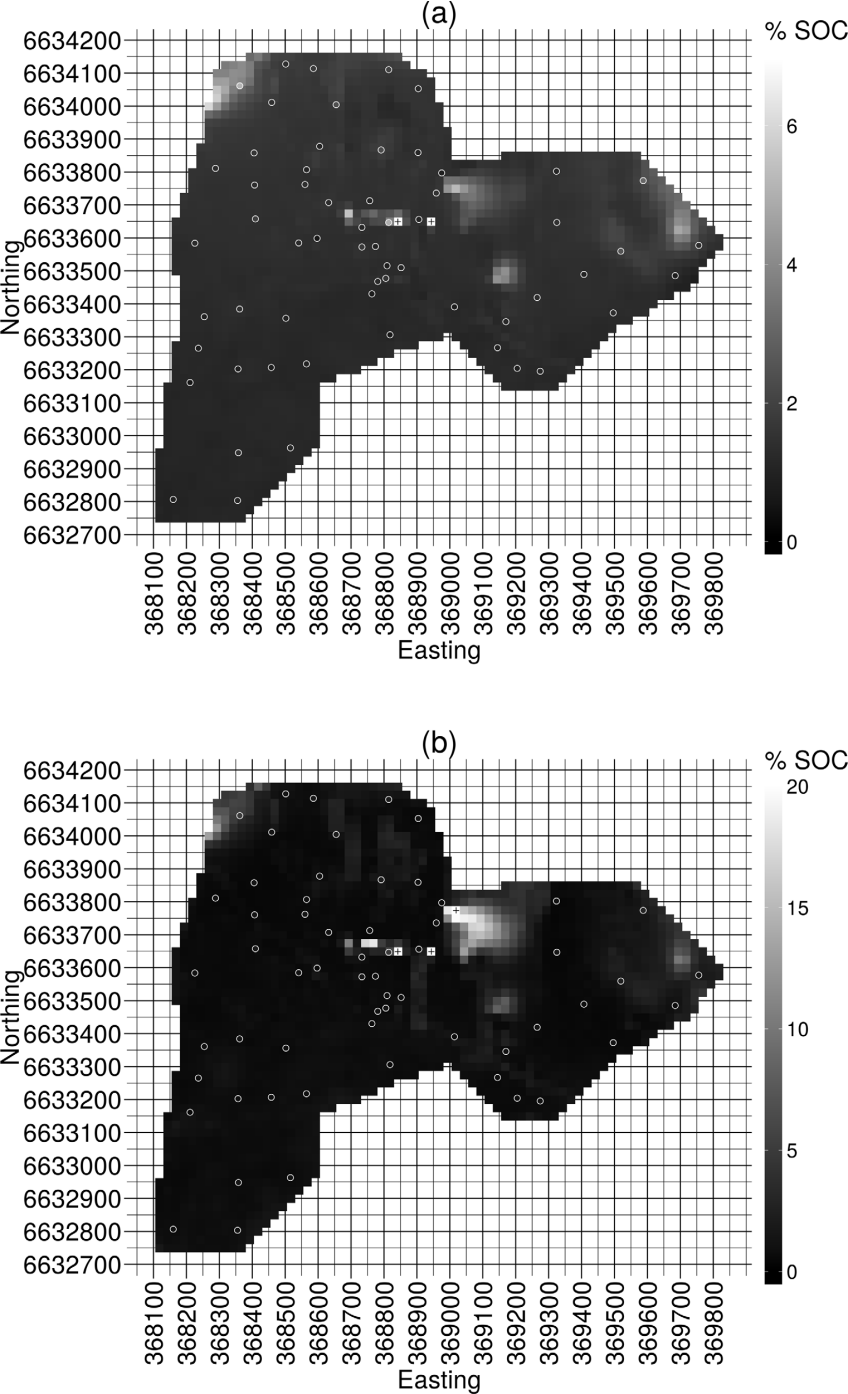
The observed soil organic carbon percentages (%SOC) at the soil core locations have been represented by the shade filling the circles located at each of the soil core sample locations. The observed %SOC values have been represented with the same grey scale as the predicted %SOC values and associated uncertainties in the rasters. **(a)** The sum of the covariate based predictions and the predictions from the model for the spatial component of the errors from the covariate based model. The more westerly pixel annotated with a vertical cross represents a predicted %SOC value of 17.92 and the more easterly pixel annotated with a vertical cross represents a predicted %SOC value of 9.54. **(b)** The uncertainty estimated to accompany the %SOC predictions. The three pixels annotated with vertical crosses represent estimates of the uncertainty associated with the model-averaged predicted %SOC values of 20.57, 21.66 and 43.66 units on the predicted %SOC scale. The estimated uncertainty of 43.66 being the most westerly of these three pixels and the estimated uncertainty of 20.57 being the most northerly of these three pixels.

As the majority of the covariates are derived from the DEM all other covariates are interpolated to the pixels of the DEM and the final prediction raster for %SOC is the result of evaluating the models at each of these pixels. The 500 selected models (each selected for one of the unique training sets) yield 500 predicted values for %SOC at every pixel in the final prediction raster. A %SOC prediction for each of these pixels is calculated via the weighted model-averaging procedure described in Section 4.1. An uncertainty estimate for these predictions is also calculated. Here the uncertainty associated with the model-averaged prediction at a pixel is quantified by the width of the interval containing the middle 95% of the predictions for that pixel. A panel of two rasters is presented in Fig 3. The areal prediction of %SOC levels across the study area plus the areal prediction of the spatial component of the errors from the covariate based modelling is presented as the top raster in Fig 3. The predictions for each pixel from the covariate based modelling are constructed by model-averaging the predictions for that pixel from the models selected by LAR exploration of the 500 unique, 35 observation training sets constructed by subsetting the 800 column design matrix. The estimate of the uncertainty associated with these predictions is presented as the bottom raster in Fig 3. The predicted spatial distribution of %SOC levels is overall quite uniform across the study site with only a few localized regions of notably elevated or depressed values. The estimated uncertainty associated with the predicted %SOC levels is relatively low across the majority of the study site with a few regions of notably elevated uncertainty. Alternative colour versions of Fig 3 are included as S1 and S2 Figs.

## 5 Discussion

This work demonstrates the suitability of LASSO modified MLR as implemented through the LAR algorithm for covariate assisted interpolation of a univariate, geostatistical response variable in a pedological context. While the case study presented here involved digital soil mapping of %SOC this analysis task occurs in a variety of pedological, ecological and environmental modelling contexts. The computational efficiency of the LAR algorithm is such that it is feasible to explore 500 unique, 35 observation subsets of a design matrix composed of 800 potential covariate terms, whereas the application of exhaustive search variable selection to this task would not have been computationally feasible. While LAR is often applied to the exploration of potential model spaces composed solely of linear main effects it may also be applied to the exploration of potential model spaces which include both polynomial terms for covariates and terms for the interactions of two or more covariates implemented through products of these terms. Efron et al. [2004] illustrate the exploration of such a model space in their simulation study which compares LAR, LAR-LASSO and Stagewise solution paths obtained from a potential model space comprised of linear main effects, interaction terms and quadratic terms. In such cases, the LAR algorithm is executed upon a design matrix that includes appropriately recentred and rescaled columns for polynomial terms and interaction terms. In the case study 63 covariates are expanded to 2205 potential covariate terms by considering polynomial terms for all covariates up to polynomial order 4 and all possible pairwise linear interaction terms. Filtering this full design matrix to enforce a MCCM between covariate pairs of 0.95 results in a design matrix comprised of 800 potential covariate terms. The L_1_ penalty in LASSO regression allows for exploration of design matrices that include such highly collinear pairs of covariates. In contrast, it would be advisable to discard a great deal more of these covariates to reduce the degree of collinearity in the design matrices examined prior to conducting the variable selection with OLS based approaches such as information criteria based stepwise variable selection. Concern regarding discarding numerous members of correlated pairs of covariates prior to conducting the variable selection appears justified in the case study. The VSEPE distributions arising from models fitted to design matrices filtered to enforce a MCCM between covariate pairs of 0.4 are more dispersed about zero than the VSEPE distributions arising from models fitted to design matrices filtered to enforce MCCM between covariate pairs of 0.95. Furthermore, it is the model-averaged predictions of the models selected from exploration of training sets constructed from this less stringently filtered design matrix that have the greatest coefficient of determination.

A key assumption of the analysis presented in this work is that correlations between covariates and the response variable do not vary across the study area. That is, spatially stationary regression coefficients are assumed in the first stage of modelling the spatial distribution of %SOC. Using spatially non-stationary linear regression coefficients could have resulted in quite similar predictive accuracies to those obtained in the modelling conducted for the case study analysis if some of the covariates varied in a spatially correlated manner. If there is spatial non-stationarity in a correlation between a covariate and some component of the response variable, this variation could well have been captured in the models presented here by the selection of a polynomial term for the covariate in question were it also varying spatially. If this were the case, it would be difficult to show one of these two interpretations to be more valid without additional information beyond that available for the case study. Given the primary objective here of interpolating the response variable, the mechanism by which this interpolation is achieved (spatially stationary coefficients of polynomial terms or spatially non-stationary coefficients of linear terms) is less important than it would be if the analysis were being conducted in an attempt to identify the pedological and edaphic processes that produced the observed distribution of %SOC.

Limitations of the analysis presented here include the interpolation of the covariates to the locations at which the response variable was observed being accomplished via separate models before the variable selection is performed. Further limitations stem from these interpolations being accomplished in a manner contingent upon the assumption of isotropic spatial dependence (see for example [63] for an explanation of this term) in the mean deviations of the covariates being realigned. Realigning the covariates by means external to the model in which variable selection is conducted is equivalent to assuming that the covariate values supplied to the variable selection process are observed without error at the locations at which the response variable was observed. However, there was uncertainty associated with both the collection of the covariates and the interpolation of the covariates to the locations at which the response variable was observed. The hierarchical Bayesian models for spatially misaligned data outlined by Banerjee et al. [2004] would be an interesting extension in this regard if these models could be extended to accomplish the variable selection task encountered in this case study. The advantage of such an approach would be a more realistic propagation of uncertainty, including the uncertainty associated with the spatial realignment of the data layers, through the model hierarchy to that associated with the final full cover areal predictions rather than the more limited cross validation based estimation of the uncertainty associated with areal prediction calculated in the analysis presented here. If this were combined with a Bayesian LASSO, where the shrinkage parameter could be assigned a hyperprior and estimated as part of the model structure, the need for cross validation would no longer be as strong but the computational challenge would likely be substantial.

Covariates related to soil water and runoff appear useful for predicting the observed distribution of %SOC (see Table 3). Given this apparent influence of water movement on the observed %SOC distribution, information regarding which catchment basin an observation was collected from could also prove useful for predicting the %SOC level associated with this observation. Namely, some catchment basins may have more %SOC moving through them than others and thus the case could be made for models that assign these catchments higher basal levels of %SOC which are then modified by the values of other covariates observed at the soil core points. Observations within a particular catchment could also be more related to other observations within this catchment than to observation from different catchments basins. Such heightened dependence among observations from the same catchment would violate the assumption of independent and identically distributed errors across all observations inherent in multiple linear regression based modelling. Thus it could be interesting to examine the utility of incorporating into the models information regarding the identity of the catchment basins from which observations were drawn and worthwhile attempting to model the covariance structure among these observations in a manner which reflects the grouping of the observations into catchment basins. Both these aims could be addressed via linear mixed effects models [64]. The effect of catchment basins within which individual observations were nested could be incorporated by random effects for each of the catchment basins while covariate effects at the soil core locations could continue to be treated as fixed effects. Such a treatment would be accompanied by a covariance structure that reflects the potentially heightened dependence among observations from the same catchment basin in the model structure. Should a larger variable selection task be feasible, random effects for all covariates could be considered in addition to the fixed effects for these covariates and the random effects for catchment basin membership (random intercept terms). The random effects for covariates provide catchment specific modifications to the slope parameters for individual covariates provided by the fixed effects. Shrinkage methods (such as LASSO and related methods) for fitting and conducting variable selection for linear mixed effects models are reviewed in Müller et al. [65]. Müller et al. [65] found that methods for implementing shrinkage on the parameters for both fixed and random effects had only been proposed in three articles at that time. These papers [66–68] use SCAD [54] or Adaptive LASSO [53] penalization and either expectation-maximization algorithm derived methods or original methods to estimate parameters. Alternatively, catchment basin effects could be incorporated into a Bayesian hierarchical (multi-level) approach via a spatial regression [17] whereby some covariates are used at the level of the geostatistical soil core observations (or at the level of the DEM pixels) in the spatial hierarchy and the covariates encoding catchment basin membership are used at the catchment basin level in the spatial hierarchy.

Linear mixed effects models also provide a means of accounting for temporal correlations among observations from multiple time periods. If we had both covariate and response observations from a couple of time periods, some from a summer survey and some from a winter survey for instance, random effects could be introduced for the different time periods and a covariance structure could be selected to account for the dependence of observations from the same time period by treating time periods as clusters of dependent observations [64]. In addition to random intercept terms and fixed effects for covariates, random effects could be introduced for covariates to explore the potential for different relationships between covariates and the response in different seasons. Linear mixed effects models also encompass methods for modelling temporal autocorrelation in time series data (also known as longitudinal data) via a variety of covariance structures [69]. Thus, if we had observations from numerous time periods, linear mixed effects models could be fitted that account for temporal dependence in the data [69].

Other penalized likelihood methods such as adaptive LASSO [53], SCAD [54] and MCP [55] could all form interesting comparisons to the LASSO modified MLR fitted with the LAR algorithm utilised in this work. Further interesting comparisons could be conducted with Bayesian LASSO [44], model-averaged Bayesian CART [45], random forests [47], boosted regression trees [49] and model-averaged Bayesian treed regression [51] with Bayesian LASSO implemented in the terminal node MLRs.

## Supporting Information

S1 Appendices Appendices to ‘Ultrahigh Dimensional Variable Selection for Interpolation of Point Referenced Spatial Data: A Digital Soil Mapping Case Study’. (PDF)

S1 Fig An alternative colour version of Fig 3. (PDF)

S2 Fig An alternative colour version of Fig 3. (PDF)

S1 Table A summary of the diversity of soil carbon modelling studies available in the literature. This table summarizes the diversity of spatial extents, land use types, geographic locations, statistical techniques and types of covariates used in such studies.
(PDF)
