# Supplementary figures and images for: Ultrahigh Dimensional Variable Selection for Interpolation of Point Referenced Spatial Data: A Digital Soil Mapping Case Study
Source: PLoS One. 2016 Sep 7;11(9):e0162489. doi: 10.1371/journal.pone.0162489 (PMC5014409; doi:10.1371/journal.pone.0162489)

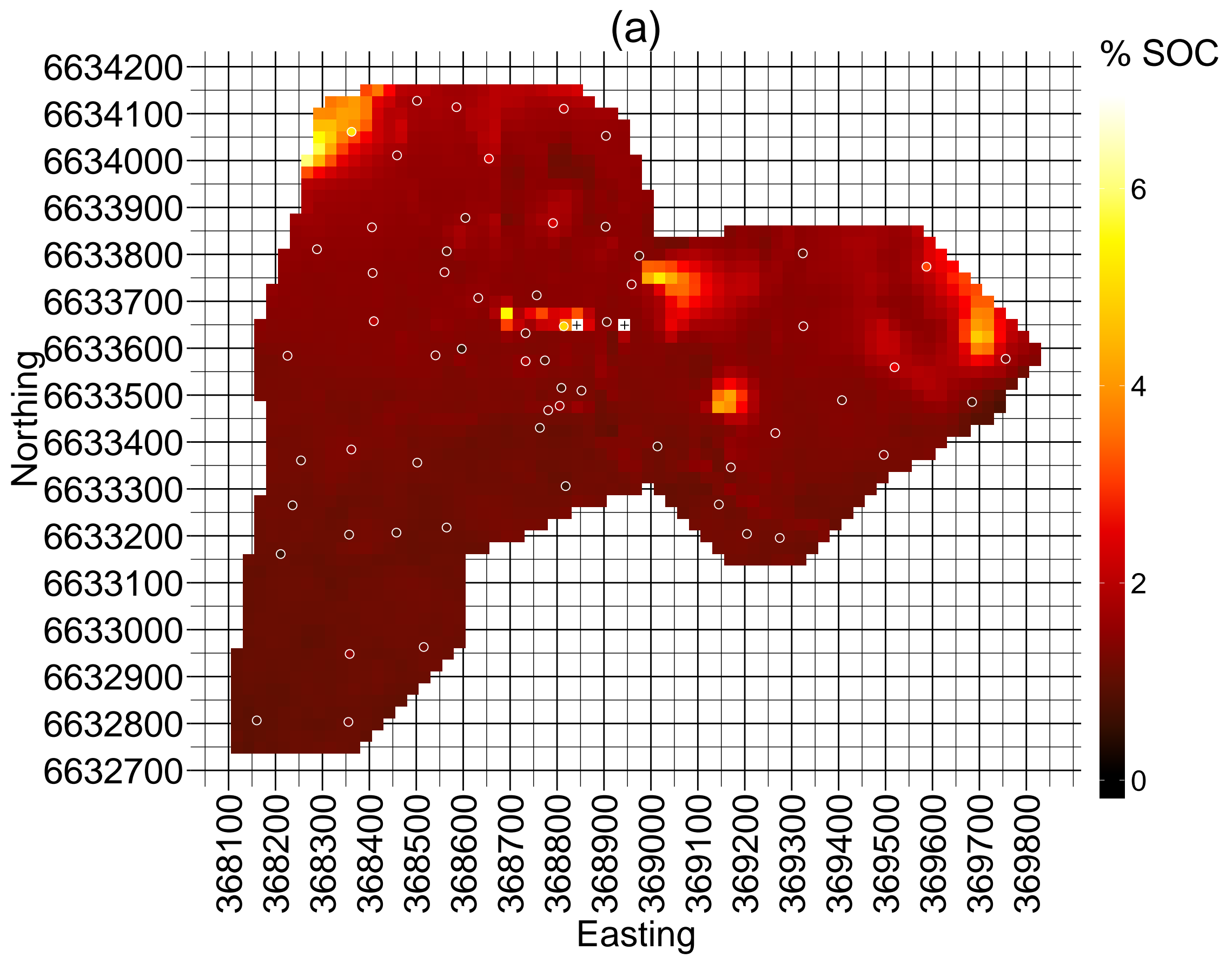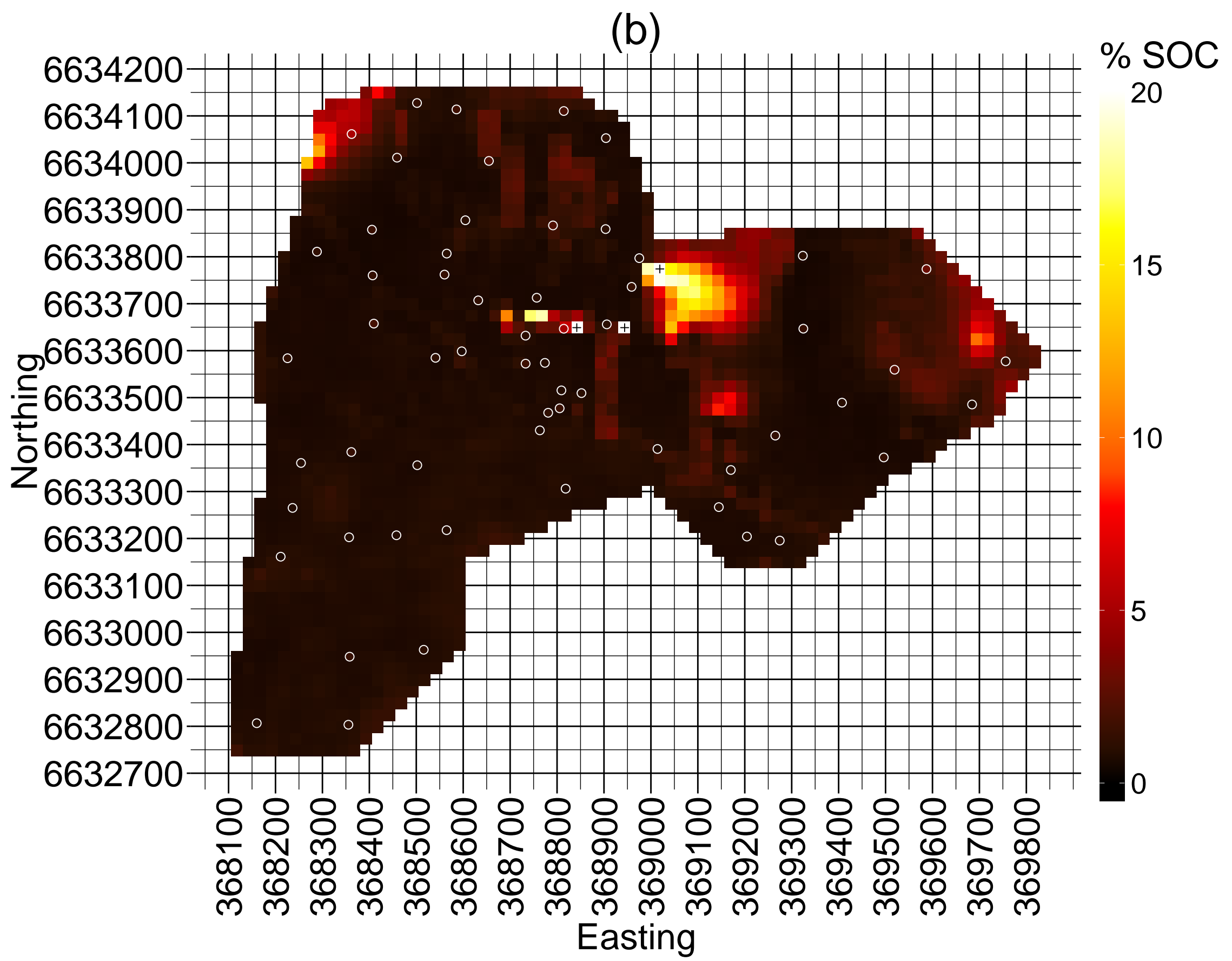

Supplement: S1 Fig — (PDF) [file pone.0162489.s002.pdf]

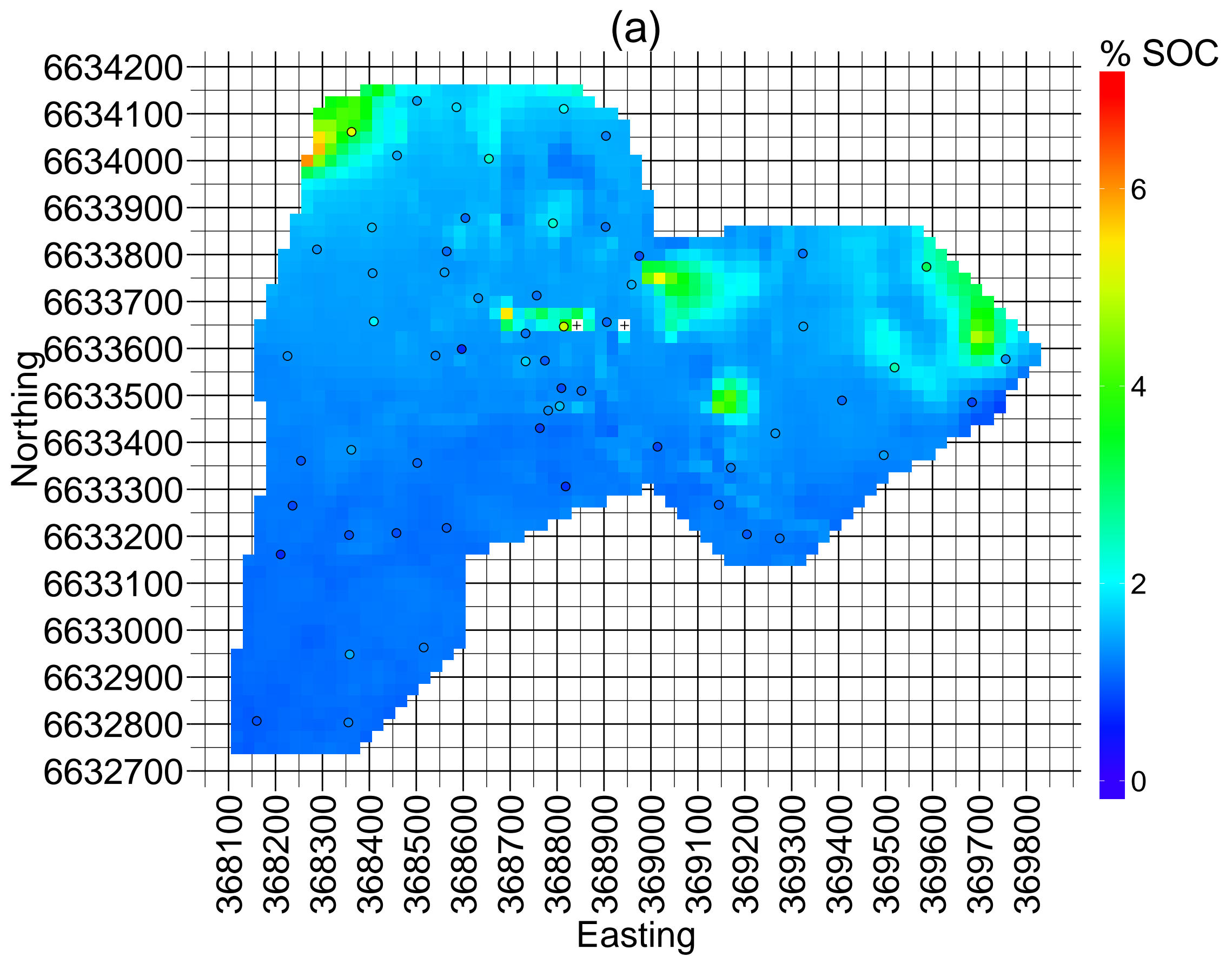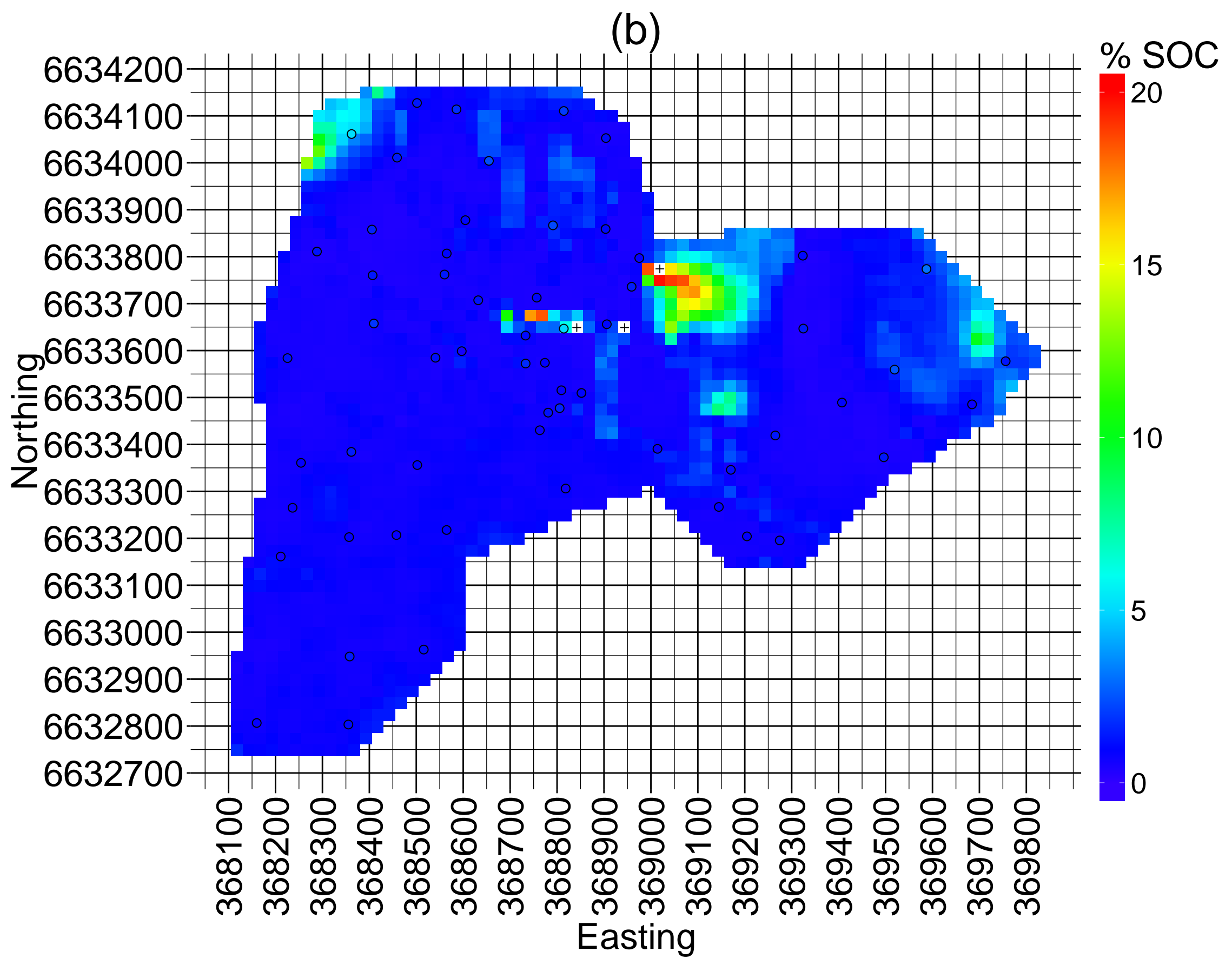

Supplement: S2 Fig — (PDF) [file pone.0162489.s003.pdf]
