# Supplementary material for: Ultrahigh Dimensional Variable Selection for Interpolation of Point Referenced Spatial Data: A Digital Soil Mapping Case Study
Source: PLoS One. 2016 Sep 7;11(9):e0162489. doi: 10.1371/journal.pone.0162489 (PMC5014409; doi:10.1371/journal.pone.0162489)
Supplement: S1 Table — This table summarizes the diversity of spatial extents, land use types, geographic locations, statistical techniques and types of covariates used in such studies. (PDF) [file pone.0162489.s004.pdf]

S1 Table

Table Supporting: Ultrahigh Dimensional Variable Selection for Interpolation of Point Referenced Spatial Data: A Digital Soil Mapping Case Study

Benjamin R. Fitzpatrick[1,2,3,*], David W. Lamb[4,2], Kerrie Mengersen[5,1,2,3]
1 Mathematical Sciences School, Queensland University of Technology (QUT), Australia
2 Cooperative Research Centre for Spatial Information (CRCSI), Australia
3 Institute for Future Environments, Queensland University of Technology (QUT), Australia
4 School of Science and Technology, University of New England (UNE), Australia
5 ARC Centre of Excellence for Mathematical and Statistical Frontiers, Queensland University of Technology (QUT), Australia
*** E-mail: Corresponding b1.fitzpatrick@qut.edu.au**

Table 1: A summary of studies modelling soil carbon with various categories of environmental data considered as covariates. A 'C' in a column for an environmental covariate category means that one or more covariate from this category was considered in the soil carbon modelling of the study in question. An 'I' in a column for a environmental covariate category means that one or more covariate from this category was found important for predicting soil carbon in the study in question.

Study	Spatial Extent	Land Use	Location	Statistical Technique	Soil Chars.	Crop/ Veg. Data	Terrain Metrics	Soil Hydrology Metrics	Geology	Land Use	Ecol./ Physiog. Region	Climate
[1]	0.4ha	Cotton	Alabama, USA	Pearson Correlation	I							
[2]	2.5 & 9ha	Grains	Westphalia, Germany	partial least squares regression	I							
[3]	4.2ha	WWBSB	Westphalia, Germany	Pearson Correlation then Regression Kriging			I	I				
[4]	5.4ha	Grains	Colorado, USA	MLR Stepwise VS			I	I				
[5]	9ha	Cotton	Alabama, USA	MLR Stepwise VS Variant	I		I					
[6]	11.2 - 28.6ha	Cotton/ Peanut	Georgia, USA	Group by Neural Network then stepwise MLR within group	I							
[7]	12.5ha	Corn/ Soy	Michigan, USA	Stepwise MLR			I					

Study	Spatial Extent	Land Use	Location	Statistical Technique	Soil Chars.	Crop/ Veg. Data	Terrain Metrics	Soil Hydrology Metrics	Geology	Land Use	Ecol./ Physiog. Region	Climate
[8]	48.7 - 65.4ha	Maize/ Soy	Nebraska, USA	Multivariate Regression Kriging	I		C					
[9]	66.3ha	Cereal/ Broadleaf	Manitoba, Canada	MLR & Stepwise VS			I	C				
This work	137ha	Native Pasture	NSW, Australia	MLR & LASSO	I	I	I	I	C			
[10]	250ha	Wheat/ Fallow	Colorado, USA	ANOVA	I							
[11]	460ha	Crop& Pasture	NSW, Australia	CUBIST	I		I	C	I			
[12]	$10km^2$	Ag. & Woods	North-West France	CUBIST		I	I	I	I	C		
[13]	$3600km^2$	Native Pasture	Mongolia, China	RF & CART	I		C	C	I	I		
[14]	$4.3*10^4km^2$	Mixed	All of Denmark	CUBIST	C		I	I	C	I	C	I
[15]	$4.3*10^4km^2$	Mixed	All of Denmark	CART	I	C	C	C	I			

Study	Spatial Extent	Land Use	Location	Statistical Technique	Soil Chars.	Crop/ Veg. Data	Terrain Metrics	Soil Hydrology Metrics	Geology	Land Use	Ecol./ Physiog. Region	Climate
[16]	$7.1*10^4 km^2$	Crop & Grasslands	Bavaria, Germany	MLR on PCA	I		I	I	C	I		C
[17]	$1.5*10^5 km^2$	Mixed	All of, Florida USA	CUBIST & Others	I	I	C	C	I	I	C	C
[18]	$6.4*10^5 km^2$	Mixed	All of France	MLR Stepwise VC	I				I	I		I
[19]	$1.1*10^5 km^2$	Mixed	Sanjiang Plain, China	GLM & Correlations	I					C		I
[20]	$7.7*10^6 km^2$	Mixed	All of Australia	CUBIST	I	I	I	C	I	C	I	I

Abbreviations & Acronyms:
C = Considered, I = Important
WWBSB = winter wheat, barley and sugar beet rotation
MLR = Multiple Linear Regression
VS = Variable Selection
LASSO = Least Absolute Shrinkage and Selection Operator
ANOVA = ANalysis Of VAriance
CART = Classification And Regression Tree
RF = Random Forests
PCA = Principal Component Analysis
GLM = Generalize Linear Model
Chars. = Characteristics

Study	Spatial Extent	Land Use	Location	Statistical Technique	Soil Chars.	Crop/ Veg. Data	Terrain Metrics	Soil Hydrology Metrics	Geology	Land Use	Ecol./ Physiog. Region	Climate

Veg. = Vegetation
Physiog. = Physiographic
Note: CUBIST is software that implements Quinlan's model tree method

References

[1] Siri-Prieto G, Reeves DW, Shaw JN, Mitchell CC. World's Oldest Cotton Experiment: Relationships between Soil Chemical and Physical Properties and Apparent Electrical Conductivity. Communications in Soil Science and Plant Analysis. 2006 Jun;37(5-6):767–786.

[2] Hbirkou C, Pätzold S, Mahlein AK, Welp G. Airborne hyperspectral imaging of spatial soil organic carbon heterogeneity at the field-scale. Geoderma. 2012;175-176:21–28.

[3] Dlugoß V, Fiener P, Schneider K. Layer-Specific Analysis and Spatial Prediction of Soil Organic Carbon Using Terrain Attributes and Erosion Modeling. Soil Science Society of America Journal. 2010;74(3):922–935.

[4] Moore ID, Gessler PE, Nielsen GA, Peterson GA. Soil Attribute Prediction Using Terrain Analysis. Soil Science Society of America Journal. 1993;57(2):443–452.

[5] Terra JA, Shaw JN, Reeves DW, Raper RL, van Santen E, Mask PL. Soil Carbon Relationships with Terrain Attributes, Electrical Conductivity, and a Soil Survey in a Coastal Plain Landscape. Soil Science. 2004;169(12):819–831.

[6] Chen F, Kissel DE, West LT, Adkins W, Rickman D, Luvall JC. Mapping Soil Organic Carbon Concentration for Multiple Fields with Image Similarity Analysis. Soil Science Society of America Journal. 2008;72(1):186.

[7] Mueller TG, Pierce FJ. Soil Carbon Maps: Enhancing Spatial Estimates with Simple Terrain Attributes at Multiple Scales. Soil Science Society of America Journal. 2003;67(1):258–267.

[8] Simbahan GC, Dobermann A, Goovaerts P, Ping J, Haddix ML. Fine-resolution mapping of soil organic carbon based on multivariate secondary data. Geoderma. 2006 Jun;132:471–489.

[9] Florinsky IV, Eilers RG, Manning GR, Fuller LG. Prediction of soil properties by digital terrain modelling. Environmental Modelling & Software. 2002;17(3):295–311.

[10] Johnson CK, Doran JW, Duke HR, Wienhold BJ, Eskridge KM, Shanahan JF. Field-Scale Electrical Conductivity Mapping for Delineating Soil Condition. Soil Science Society of America Journal. 2001;65:1829–1837.

[11] Miklos M, Short MG, McBratney AB, Minasny B. Mapping and comparing the distribution of soil carbon under cropping and grazing management practices in Narrabri, north-west New South Wales. Australian Journal of Soil Research. 2010;48:248–257.

[12] Lacoste M, Minasny B, McBratney A, Michot D, Viaud V, Walter C. High resolution 3D mapping of soil organic carbon in a heterogeneous agricultural landscape. Geoderma. 2014;213:296–311.

[13] Wiesmeier M, Barthold F, Blank B, Kögel-Knabner I. Digital mapping of soil organic matter stocks using Random Forest modeling in a semi-arid steppe ecosystem. Plant and Soil. 2011 May;340(1-2):7–24.

[14] Adhikari K, Hartemink AE, Minasny B, Kheir RB, Greve MB, Greve MH. Digital Mapping of Soil Organic Carbon Contents and Stocks in Denmark. PLOS ONE. 2014;9(8):e105519.

[15] Kheir RB, Greve MH, Bø cher PK, Greve MB, Larsen R, McCloy K. Predictive mapping of soil organic carbon in wet cultivated lands using classification-tree based models: The case study of Denmark. Journal of Environmental Management. 2010;91(5):1150–1160.

[16] Wiesmeier M, Hübner R, Barthold F, Spörlein P, GeußU, Hangen E, et al. Amount, distribution and driving factors of soil organic carbon and nitrogen in cropland and grassland soils of southeast Germany (Bavaria). Agriculture, Ecosystems and Environment. 2013;176:39–52.

[17] Xiong X, Grunwald S, Myers DB, Kim J, Harris WG, Comerford NB. Holistic environmental soil-landscape modeling of soil organic carbon. Environmental Modelling & Software. 2014;57:202–215.

[18] Meersmans J, Martin MP, Lacarce E, De Baets S, Jolivet C, Boulonne L, et al. A high resolution map of French soil organic carbon. Agronomy for Sustainable Development. 2012;32(4):841–851.

[19] Mao DH, Wang ZM, Li L, Miao ZH, Ma WH, Song CC, et al. Soil organic carbon in the Sanjiang Plain of China: storage, distribution and controlling factors. Biogeosciences. 2015;12(6):1635–1645.

[20] Rossel RAV, Webster R, Bui EN, Baldock JA. Baseline map of organic carbon in Australian soil to support national carbon accounting and monitoring under climate change. Global Change Biology. 2014;20:2953–2970.
